# Evidence of epistatic suppression of repeat fruiting in cultivated strawberry

**DOI:** 10.1186/s12870-019-1984-7

**Published:** 2019-09-05

**Authors:** K. S. Lewers, P. Castro, J. F. Hancock, C. K. Weebadde, J. V. Die, L. J. Rowland

**Affiliations:** 1 USDA-ARS, Genetic Improvement of Fruits and Vegetables Laboratory, Building 010A BARC- West, 10300 Baltimore Ave., Beltsville, MD 20705-2350 USA; 2 Department of Genetics, Escuela Técnica Superior de Ingenieros Agrónomos, Edificio Gregor Mendel (C-5), Campus de Rabanales, University of Cordoba, 14071 Córdoba, Spain; 3 Department of Horticulture, A342C Plant and Soil Sciences Building, Michigan State University, East Lansing, MI 48824-1325 USA; 4 Department of Plant, Soil and Microbial Sciences, A384-D Plant and Soil Sciences Building, Michigan State University, East Lansing, MI 48824-1325 USA

**Keywords:** Marker-assisted breeding, Non-additive, Dayneutral, Perpetual flowering, Everbearing, Remontant

## Abstract

**Background:**

Consumers purchase fresh strawberries all year long. Extending the fruiting season for new strawberry cultivars is a common breeding goal. Understanding the inheritance of repeat fruiting is key to improving breeding efficiency. Several independent research groups using multiple genotypes and analytic approaches have all identified a single genomic region in strawberry associated with repeat fruiting. Markers mapped to this region were used to evaluate breeding parents from the United States Department of Agriculture – Agricultural Research Service (USDA-ARS) strawberry breeding program at Beltsville, Maryland.

**Results:**

Markers mapped to repeat fruiting identified once-fruiting genotypes but not repeat-fruiting genotypes. Eleven of twenty-three breeding parents with repeat-fruiting marker profiles were actually once fruiting, indicating at least one additional locus acting epistatically to suppress repeat fruiting. Family segregation ratios could not be predicted reliably by the combined use of parental phenotypes and marker profiles, when using a single-gene model. Expected segregation ratios were calculated for all phenotypic and marker-profile combinations possible from the mapped locus combined with a hypothetical dominant or recessive suppressor locus. Segregation ratios specific to an epistatic suppressor acting on the mapped locus were observed in four families. The segregation ratios for two families were best explained by a dominant suppressor acting on the mapped locus, and, for the other two, by a recessive suppressor. Not all of the observed ratios could be explained by one model or the other, and when multiple families with a common parent were compared, there was no predicted genotype for the common parent that would lead to all of the observed segregation ratios.

**Conclusions:**

Considering all lines of evidence in this study and others, repeat-fruiting in commercial strawberry is controlled primarily by a dominant allele at a single locus, previously mapped by multiple groups. At least two additional genes, one dominant and one recessive, exist that act epistatically to suppress repeat fruiting. Environmental effects and/or incomplete penetrance likely affect phenotype through the suppressor loci, rather than the primary mapped locus. One of the dominant suppressors acts only in the first year, the year the plant is germinated from seed, and not after the plant has experienced a winter.

**Electronic supplementary material:**

The online version of this article (10.1186/s12870-019-1984-7) contains supplementary material, which is available to authorized users.

## Background

Strawberries (*Fragaria* ×*ananassa* Duchesne ex Rozier) are an economically important crop in the United States (US). At 2.3 billion US dollars, the US 2014 strawberry crop was third-most valuable after grapes (6.3 billion USD) and apples (3.5 billion USD) among noncitrus fruits [1]. They also are a valuable crop for growers at a national average of $110,051 gross per hectare, ranging from $20,626 per hectare in Oregon to $119,816 in California 1). The high value of strawberries is due in part because of their popularity. Strawberries ranked as the fifth most purchased fresh-market fruit in the US [2], with annual per capita consumption increasing steadily from 2.1 to 3.6 kg from 2002 to 2013 [3]. Strawberries are highly perishable and cannot be stored fresh for long periods [4], yet consumers purchase fresh strawberries all year long. To enable year-round sales, strawberries are grown in a wide range of climates and production systems. Although most US production is on the West Coast, winter strawberries are grown primarily in Florida, and local strawberries are grown in nearly every state for direct sale.

Several strawberry breeding programs have responded to the demand for year-long production by including extended fruiting in their breeding goals. Breeders and other researchers around the world refer to plants with extended flowering and fruiting periods as day neutral, long day, everbearing, perpetual fruiting, remontant, and/or repeat fruiting. It is not clear if these are indeed the same trait controlled by the same gene or genes and affected similarly by environmental conditions, as no study has been conducted using sufficient diversity of genotypes and environments to make this determination.

Cultivated strawberries are sold in the US as two primary types, according to their flowering and fruiting habit: short day (SD) and day neutral (DN), often spelled “dayneutral” in catalogs and on web pages. Short-day genotypes, also called June bearers in northern states, initiate flower buds either under short day-lengths (< 14 h of day length) or at temperatures below 15 °C. These plants flower only once a year and, therefore, have only one harvest period. “Day-neutral” genotypes were reported to be photoperiod insensitive, initiating flowers under any photoperiod condition, as long as temperatures are moderate (below 30/26 °C day/night) [5–7]. Continued production was at least as dependent on temperature [6, 8–10], so it is inappropriate, outside the industry, to refer to plants with the trait as “dayneutral plants”. “Repeat fruiting” is a term that doesn’t imply an understanding of the environmental or physiological control of the trait and is more widely understood than “remontant”.

Because knowledge of the inheritance of any trait is useful to improving breeding efficiency, several genetic studies have addressed the inheritance of repeat fruiting. Some consider repeat fruiting to be controlled by a single gene in cultivated strawberry, either recessive [11] or dominant [12–15]. However, evidence has been provided that the number of genes controlling repeat fruiting in octoploid strawberry varies by genotype [15]. Some quantitative trait loci (QTL) mapping projects identified multiple QTL associated with repeat fruiting quantified as number of weeks of flowering [9, 16], although some early attempts used mapping software that did not yet have a way to test for significance of QTL, and some “false” QTL may have been reported. The effect of environment on the expression of repeat fruiting was highlighted by evaluating a single mapping population, clonally propagated, in several US states: Michigan, Minnesota, Oregon, California, and Maryland [9]. Several of the progeny were repeat fruiting in milder climates of California and Oregon, but once fruiting in hot bright Maryland. Two studies [16, 17] evaluated repeat fruiting both quantitatively and qualitatively in single populations, mapping the trait to the same genomic region with both approaches. Multiple researchers using different mapping populations identified the same single locus on the same linkage group (LG) in Homoeologous Group IV (HGIV) with the dominant allele conferring repeat fruiting [18, 23–25]. These mapping studies had no parents in common but may have all had a common progenitor for the repeat-fruiting trait. This progenitor, a wild *F. virginiana* ssp. *glauca* (Wats.) Staudt strawberry plant collected in the Wasatch Mountains, south of Salt Lake City, UT, has been used as the source of repeat fruiting to produce new cultivars in many breeding programs [26].

The objective of this study was to determine if molecular markers linked to this collectively identified repeat-fruiting genomic region could correctly predict phenotypes and segregation ratios in breeding families in the USDA-ARS strawberry breeding program pool at Beltsville, MD. This breeding program produced ‘Delmarvel’ [27], which was used as the once-fruiting parent pollinated by ‘Selva’, a repeat-fruiting parent, to map repeat fruiting [18]. And the Beltsville program also produced ‘Tribute’ [28], the repeat-fruiting parent, which was pollinated by ‘Honeoye’, and used in other studies to map repeat fruiting [9, 17]. The pedigree for ‘Tribute’ goes back to the wild *F. virginiana* ssp. *glauca* plant attributed as the source of repeat fruiting in cultivars around the world [28]. Therefore, it is reasonable to assume the mapped repeat-fruiting genomic region, and the Beltsville program breeding pool originated from the same source.

## Results

### Developing new simple-sequence repeat (SSR) markers for the repeat-fruiting region

Previously, the SSR marker most closely associated with repeat fruiting was FxaACA02I08C -145S, 14 cM from the trait [18]. Therefore, the primer sequences of FxaACA02I08C were used to search for regions with sequence similarity in the *F*. ×*ananassa* reference genome. The approximate string-matching algorithm found sequence similarities in the scaffold FANhyb_rscf00000019.1, which was selected for further analysis. To identify other regions that could be used as a target from which to design more primers for repeat fruiting, other SSR markers (Table 1) previously linked to the trait [17, 18] were used to search the *F.* ×*ananassa* reference genome. Using this approach, seven different primers were located to seven scaffolds: six scaffolds contained sequence similarities to three primers, and one scaffold contained sequence similarities to two primers. In summary, the approximate string-matching algorithm found seven scaffolds that could potentially be from the mapped repeat-fruiting genomic region. Those seven scaffolds were searched for SSR motifs, and 36 primer pairs were developed to amplify the most promising repeat regions (Additional file 1: Table S1).

**Table 1:** List of SSR markers linked to repeat-fruiting in cultivated strawberry. “SS” indicates the primer sequences were used individually in sequence-similarity searches against the cultivated strawberry reference genome. “BP” means the primer pairs were used in PCR reactions with 32 breeding parents of known flowering phenotype in the USDA-ARS Beltsville strawberry breeding program

SSR name	Use	Primer sequences	Reference
ChFaM011	SS, BP	F: TCCTCTCCTTCTTTCCCTTCA	Zorrilla-Fontanesi et al. 2011 [19]
		R: CGAGATCTCCCGAGACTGAG	
EMFvi136	SS	F: GAGCCTGCTACGCTTTTCTATG	Sargent et al. 2003 [20]
		R: CCTCTGATTCGATGATTTGCT	
F.v.D3	SS, BP	F: CAGGATCGTTCTTGCTAGTG	Spigler et al. 2010 [21]
		R: GTTTCTGTTTTCTGGGGTTTGAATA	
CX661225	SS	F: GCTCTCCTCCTCCGTCTCTT	Spigler et al. 2008 [22]
		R: GTTTAGCTTCTCCGCTGACG	
ChFaM148	SS, BP	F: CCCTCCATCAAAGCCAGTT	Zorrilla-Fontanesi et al. 2011 [19]
		R: CATTAGACCCCGACTTGTCA	
ChFaM148A	SS	F: GGTTCTGCGCCACATAGCTA	Current study
		R: CGTATGAGGACGGGGAGGTA	
ChFaM148B	SS	F: TTGTGCTGCCCTCCATCAAA	Current study
		R: TGCACTCTTCCATGGCTTCC	
ChFaM148C	SS, BP	F: GTAGAGATCGATGTATGGTACGT	Current study
		R: GGATGTCCAATTTGGTTCTGTT	
ChFaM148D	SS	F: GCGACTGAGGCGTACATACT	Current study
		R: CGATCAACATCTTGGCAACCC	
FxaACA02I08C	SS, BP	F: TAGCAGAAGCATTTCACTCCCTCC	Honjo et al. 2016 [23]
		R: GAATGGCATTTGAGAAATGAGAGCA	
FANhyb19.1–15	BP	F: TTCTCCATCTTCACATTTCTG	Current study
		R: TGCTCTCTTTTGCTAAGTACG	
FANhyb19.1–4	BP	F: GGTCTGGATTTAGGAGAGAAA	Current study
		R: TCTTCTTCTTTCATCAGAAGC	
FANhyb19.1–1	BP	F: GCAACTTCTCAACTTTAGCAC	Current study
		R: CGTGATGAGTTCAAATTAAGG	
FANhyb19.1–6	BP	F: GGTTTAGGAGAGAAACCAATC	Current study
		R: GATTACACTCGCACACTTCTC	
FANhyb19.1–3	BP	F: CGCAGCACCAGTCTTTCATG	Current study
		R: AACCCTCCTCCTCCTCCTTC	
FxaAGA01G05C	BP	F: CATTTATCACTGTTTGGCGTTCCC	Honjo et al. 2016 [23]
		R: CAAAACACCCACGATTATACGGGT	
FxaAGA02N04C	BP	F: AACAAATTTCAAACCTGAGGAGCG	Honjo et al. 2016 [23]
		R: AACAGTGTTGCAGTTTCATCCGAA	
Bx056	BP	F: GGTTACTGGCTCTGCTTGGA	Perrotte et al. 2016 [24]
		R: CACAATTGAGATCGAGCAACA	
Bx059	BP	F: GACGTTGACCATGACAGAGC	Perrotte et al. 2016 [24]
		R: CCCAAAGAAAGCCCAACATA	
Bx064	BP	F: GGGGAGGTGAAACTGTGAAA	Perrotte et al. 2016 [24]
		R: TGCAATCTTTGGGAGAGAGAA	
Bx083	BP	F: ACGTGCCTTAGCGGATCATA	Perrotte et al. 2016 [24]
		R: CCTAGGTCGGGATCTCAGAA	
Bx089	BP	F: CACCAAAGATGACTGCTGGA	Perrotte et al. 2016 [24]
		R: TAAACAGCCCCTGAATCCAA	
Bx215	BP	F: CAATTTCCCGCCAAAAGTAA	Perrotte et al. 2016 [24]
		R: GTTGGAGCTTCGAGCAAGTT	
Bx250	BP	F: GGCATTTCCGCAGATAAAAA	Perrotte et al. 2016 [24]
		R: AACCTCCTCGTGTTTGATGC	

**Fig. 1:**
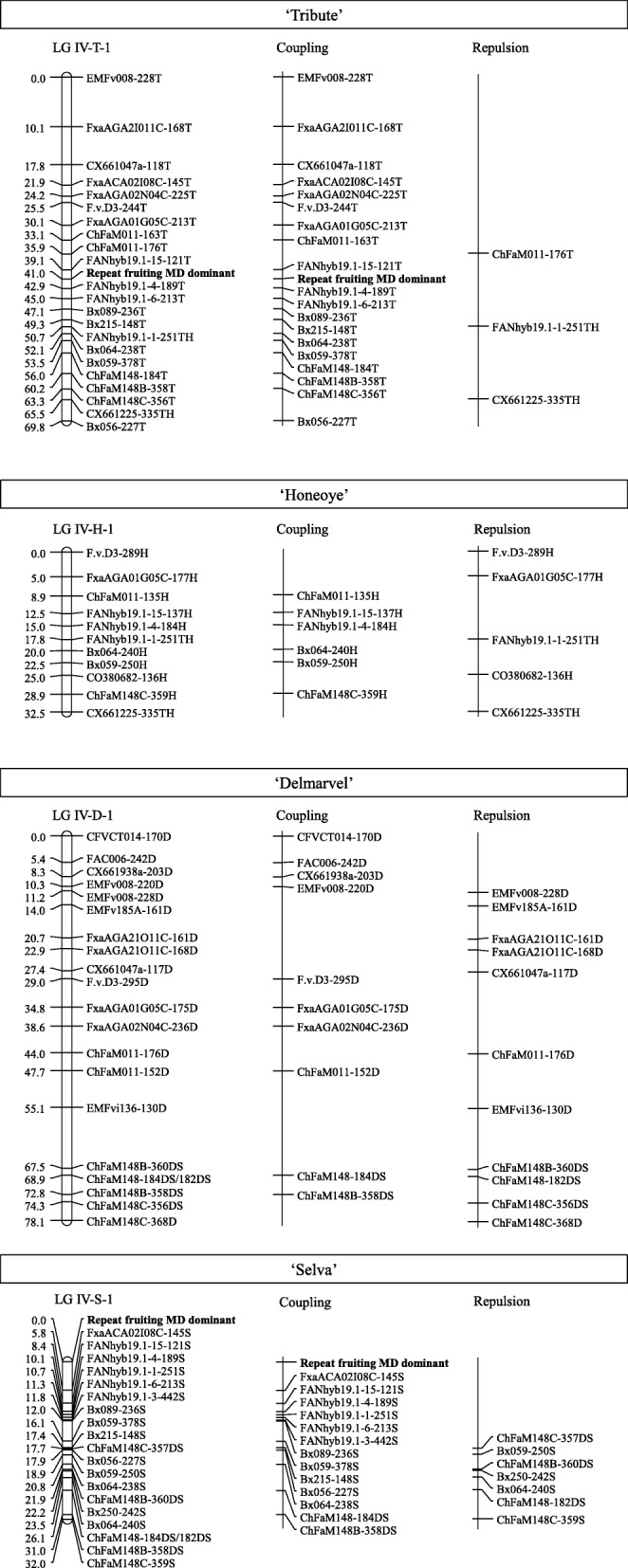
Molecular markers linked with repeat fruiting in commercial strawberry (*F.* ×*ananassa*). Maps were constructed with two F_1_ populations segregating 1:1 for repeat fruiting and once fruiting: ‘Tribute’ × ‘Honeoye’ and ‘Delmarvel’ × ‘Selva’. The name of each parent is shown in a box. Under the name of each parent is shown the linkage group (left) and location of the repeat-fruiting trait in bold text, the markers linked to the repeat-flowering trait in coupling (center), and in repulsion (right)

Six (FANhyb19.1–7, FANhyb19.1–8, FANhyb19.1–9, FANhyb19.1–12, FANhyb45.1–5, FANhyb758.1–3) out of the 36 primer pairs designed did not produce any amplification products. Of the 30 primer pairs that amplified a product from the mapping parents, three (FANhyb19.1–5, FANhyb611.1–1 and FANhyb682.1–2) were polymorphic only in the ‘Delmarvel’ × ‘Selva’ population, two (FANhyb19.1–13 and FANhyb64.1–1) were polymorphic only in the ‘Tribute’ × ‘Honeoye’ population, and 13 were polymorphic in both populations. These primer pairs amplified 61 products in the ‘Delmarvel’ × ‘Selva’ population, but only 33 of them fit acceptable segregation ratios for mapping and were used to construct the linkage map. Products from primer pairs FANhyb19.1–1, FANhyb19.1–3, FANhyb19.1–4, FANhyb19.1–6 and FANhyb19.1–15 mapped to the region associated with repeat fruiting in HGIV, whereas product sizes from primer pairs FANhyb611.1–1, FANhyb682.1–1, FANhyb45.1–1, FANhyb45.1–2, FANhyb758.1–1, FANhyb758.1–4, FANhyb64.1–1, FANhyb64.1–2, FANhyb64.1–4 mapped to other HG not associated with repeat fruiting (Additional file 2: Table S2). FANhyb19.1–15-121S and FANhyb19.1–4-189S were the closest to the repeat-fruiting region at 8.4 and 10.1 cM, respectively (Fig. 1). The amplicons that mapped to HG other than HGIV were not used to genotype the ‘Tribute’ × ‘Honeoye’ population. A total of 32 products were amplified using eight primer pairs with ‘Tribute’ and ‘Honeoye’, 12 of which fit the expected segregation ratios, when used with the population, and were used for linkage analysis. Four of these products mapped to the region associated with repeat fruiting (HG IV of ‘Tribute’; Fig. 1) and the remainder mapped to HG IV, but in LGs not associated with the trait. FANhyb19.1–15-121 T and FANhyb19.1–4-189 T were the closest markers at 1.9 cM from repeat fruiting.

The eight SSR primer pairs linked to repeat fruiting in the ‘Capitola’ × CF1116 mapping population [24] amplified 59 products in the ‘Delmarvel’ × ‘Selva’ population (34 fit expected segregation ratios) and 55 products in the ‘Tribute’ × ‘Honeoye’ population (31 fit expected segregation ratios). All these markers mapped to HGIV. Bx089–236S and Bx089–236 T were the closest of this group of markers at 12 and 6.1 cM from repeat fruiting in HGIV of ‘Selva’ and ‘Tribute’, respectively (Fig. 1). Bx089 was one of the two markers most closely linked to repeat fruiting in the ‘Capitola’ × CF1116 mapping population [24].

Based on the results of the linkage maps from ‘Delmarvel’ × ‘Selva’ and ‘Tribute’ × ‘Honeoye’ populations (Fig. 1), 19 SSR markers closely linked to repeat fruiting were selected to test parents of breeding families and commercial cultivars (Table 1).

### Comparing breeding parent phenotypes and marker profiles

A total of 32 parental strawberry genotypes, plus the mapping parents and other breeding selections, were characterized with 19 markers linked to repeat fruiting (Table 2, Additional file 3: Table S3). For all parents, regardless of flowering pattern, at least four primer pairs amplified a product associated with repeat fruiting. For one repeat-fruiting parent, B2411RB, all primer pairs amplified the product associated with repeat fruiting. If all or all but one or a few primer pairs amplified a product associated with repeat fruiting, especially those with the highest Kruskal-Wallis test value, that was considered a strong indication that parent should have a repeat-fruiting phenotype. For 14 of the parents, all but one primer pair, Bx250–242, amplified a product associated with repeat fruiting in mapping, yet only half of these had repeat-fruiting phenotypes in the field in multi-year testing; thus, the molecular profile associated with repeat fruiting did not reliably predict the repeat-fruiting phenotype in the field. Bx250–242 did not map close to repeat fruiting in the ‘Delmarvel’ × ‘Selva’ population and did not map in the ‘Tribute’ × ‘Honeoye’ population.

**Table 2:** Observed and predicted phenotypes, marker profiles, and segregation ratios of strawberry (*F*. ×*ananassa*) breeding parents and families at the USDA-ARS, Beltsville, MD

Breeding families 2016			No. progeny			Observed parental phenotype			Parental phenotype predicted by marker profile			Phenotype-predicted segregation	Marker-predicted segregation, single-locus model	Simplest best-fit to observed segregation	
M^a^	×	P	All	N	F	M	×	P	M	×	P			Ratio	P > ×2
B2396	×	B1031	104	42	62	jb	×	jb	RB	×	RB	N	F or 1 N:3F	7 N:9F or 3 N:5F	0.4890 or 0.5434
B1776RB	×	“	104	35	69	RB	×	jb	jb or RB	×	RB	F or 1:1	F, 1:1, or 1 N:3F	3 N:5F	0.4178
B2483	×	“	104	72	32	jb	×	jb	jb or RB	×	RB	N	F, 1:1, or 1 N:3F	13 N:3F, 3 N:1F, 5 N:3F	0.1742, 0.1742, 0.1562
B2475	×	“	104	87	17	jb	×	jb	RB	×	RB	N	F or 1 N:3F	7 N:1F	0.2356
B2474	×	“	104	104	0	jb	×	jb	RB	×	RB	N^b^	F or 1 N:3F	N	0
B2326	×	B1543RB	114	75	39	jb	×	RB	jb or RB	×	RB^b^	F or 1:1	F, 1:1, or 1 N:3F	5 N:3F	0.4682
B1082RB	×	B1580	114	55	59	RB	×	jb	RB^b^	×	jb or RB	F or 1:1^b^	F, 1:1^b^, or 1 N:3F	1 N:1F or 7 N:9F	0.7079 or 0.3332
B1788	×	“	124	66	58	RB	×	jb	jb or RB	×	jb or RB	F or 1:1^b^	N, F, 1:1^b^, or 1 N:3F	9 N:7F or 1 N:1F	0.4972 or 0.4725
B2075	×	B1776RB	120	63	57	jb	×	RB	jb or RB	×	jb or RB	F or 1:1^b^	N, F, 1:1^b^, or 1 N:3F	9 N:7F or 1 N:1F	0.4076 or 0.5839
B2065	×	B1788	120	34	86	jb	×	RB	jb^b^	×	jb or RB	F or 1:1	N, F, or 1:1	1 N:3F	0.3991
B1540RB	×	B1806	124	28	96	RB	×	jb	RB^b^	×	RB	F or 1:1	F or 1 N:3F^b^	1 N:3F	0.5338
B1547RB	×	“	129	30	99	RB	×	jb	RB^b^	×	RB	F or 1:1	F or 1 N:3F^b^	1 N:3F	0.6473
B1846RB	×	“	128	34	94	RB	×	jb	RB^b^	×	RB	F or 1:1	F or 1 N:3F^b^	1 N:3F	0.6831
B1543RB	×	“	130	97	33	RB	×	jb	RB^b^	×	RB	F or 1:1	F or 1 N:3F	13 N:3F or 3 N:1F	0.9193 or 0.9193
B2409RB	×	“	90	41	49	RB	×	jb	RB^b^	×	RB	F or 1:1^b^	F or 1 N:3F	1 N:1F or 7 N:9F	0.3991 or 0.7299
B1788	×	“	130	59	71	RB	×	jb	jb or RB	×	RB	F or 1:1^b^	F, 1:1^b^, or 1 N:3F	1 N:1F or 7 N:9F	0.2926 or 0.7071
B2325	×	“	130	60	70	RB	×	jb	RB^b^	×	RB	F or 1:1^b^	F or 1 N:3F	1 N:1F or 7 N:9F	0.3805 or 0.5806
B2411RB	×	“	119	63	56	RB	×	jb	RB^b^	×	RB	F or 1:1^b^	F or 1 N:3F	9 N:7F or 1 N:1F	0.4669 or 0.5211
B2400	×	“	119	93	26	jb	×	jb	jb^b^	×	RB	N	F or 1:1	13 N:3F or 3 N:1F	0.4273 or 0.4273
B2484	×	“	116	91	25	jb	×	jb	jb^b^	×	RB	N	F or 1:1	13 N:3F or 3 N:1F	0.3911 or 0.3911
B2488	×	“	120	100	20	jb	×	jb	jb or RB	×	RB	N	F, 1 N:1F, or 1 N:3F	7 N:1F	0.1675
B2480	×	B1820	119	93	26	jb	×	jb	jb^b^	×	jb^b^	N	N	13 N:3F or 3 N:1F	0.4273 or 0.4273
B2473	×	“	120	116	4	jb	×	jb	jb^b^	×	jb^b^	N	N	15 N:1F	0.1869
B2072	×	B2325	56	37	19	jb	×	RB	RB	×	RB^b^	F or 1:1	F or 1 N:3F	13 N:3F, 3 N:1F, 5 N:3F, 9 N:7F	0.1228, 0.1228, 0.5809, 0.1385
B2079	×	B2396RB	120	70	50	jb	×	jb	RB	×	RB	N	F or 1 N:3F	5 N:3F or 9 N:7F	0.3458 or 0.6455
B2072	×	B2411	130	82	48	jb	×	RB	RB	×	RB^b^	F or 1:1	F or 1 N:3F	5 N:3F or 9 N:7F	0.8919 or 0.1166
B2476	×	B793	130	81	49	jb	×	jb	jb or RB	×	RB	N	F, 1 N:1F, or 1 N:3F	5 N:3F or 9 N:7F	0.9639 or 0.1638
B2479	×	“	130	105	25	jb	×	jb	jb	×	RB	N	F or 1:1	13 N:3F or 3 N:1F	0.1287 or 0.1287
B1547RB	×	Strawberry Festival	120	6	114	RB	×	jb	RB^b^	×	jb^b^	F or 1:1	F or 1:1	1 N:15F	0.5716
B2396	×	“	137	9	128	jb	×	jb	RB	×	jb^b^	N	F or 1:1	1 N:15F	0.8773
B1543RB	×	“	120	37	83	RB	×	jb	RB^b^	×	jb^b^	F or 1:1	F or 1:1	3 N:5F or 1 N:3F	0.1314 or 0.1400

^a^M - Maternal; P - Paternal; jb - Once fruiting; RB- repeat fruiting; N - Did not flower the first year; F - Flowered the first year; ^b^ - Predicted phenotype or segregation ratio fit observed phenotype or segregation ratio

Six markers were most closely associated with repeat fruiting in the ‘Tribute’ × ‘Honeoye’ population when using the Kruskal-Wallis nonparametric test of association between each marker and the number of weeks of flowering (a continuous trait): FANhyb19.1–15-121, FANhyb19.1–4-189, Bx215–148, ChFaM148–184, Bx064–238, and Bx089–236. Examining the marker phenotypes of the breeding parents, regardless of flowering pattern, FANhyb19.1–15-121 and FANhyb19.1–4-189 were either both present, or both absent, indicating very close genomic proximity to each other (Additional file 3: Table S3). Both markers were derived from the same scaffold, FANhyb_rscf00000019.1, (Additional file 1: Table S1) and were 18,399 base pairs apart. All nine of the parents with a repeat-fruiting phenotype had the markers FANhyb19.1–15-121 and FANhyb19.1–4-189, and all but two had the marker Bx215–148. Previously, the presence of both Bx083_206 and Bx215_131, only 7.3 cM apart, was hypothesized to flank the gene controlling repeat fruiting, but predicted repeat fruiting in only 14 of the 17 genotypes with a repeat-fruiting phenotype [24]. In the current study, for seven parents, the markers FANhyb19.1–15-121 and FANhyb19.1–4-189 were both present (indicating repeat fruiting), and the marker Bx215–148 was absent (no amplification product indicates no repeat fruiting). In this situation, three parents were repeat fruiting and four were once fruiting. Again, the presence of the markers FANhyb19.1–15-121 and FANhyb19.1–4-189 were not a reliable predictor of repeat fruiting in the breeding parents. It also appeared that the repeat-fruiting gene may be located between the region covered by these two markers and the marker Bx215–148. Bx215–131 was one of two markers previously linked closely to repeat fruiting [24], and much of the amplicon size difference was most likely due to the primer modifications made to save costs and improve adenylation reliability in the current study. The primer sequences of the Bx215 markers did not have sequence similarities in the scaffold FANhyb_rscf00000019.1. The Bx215 primer sequences instead had sequence similarity in scaffold FANhyb_rscf00000331.1. These results suggest that both scaffolds could be part of the same chromosome, or at least the same homoeologous group, and underscore the need for additional efforts to fill gaps and connect scaffolds to realize the potential offered by a “sequenced” genome.

Although the presence, in a breeding parent, of a marker genetically linked with repeat fruiting did not indicate whether the breeding parent would have the repeat-fruiting phenotype in the field, the absence of at least some markers reliably indicated that a plant would have the once-fruiting phenotype. If the markers FANhyb19.1–15-121, FANhyb19.1–4-189, and Bx215–148 were all absent, as in eight of the breeding parents, the phenotype in the field was once fruiting. This is consistent with a finding of 47 molecularly characterized “seasonal-flowering” genotypes missing both Bx083_206 and Bx215_131 [24]. Likewise, only once-fruiting phenotypes could be identified using SNP markers [25]. And, although a 100% negative predictive value was observed for Bx215_128, only an 85% positive predictive value was observed [29]. In the current study, for one of the once-fruiting selections, the markers FANhyb19.1–15-121 and FANhyb19.1–4-189 were both absent (no amplification product indicates once fruiting), whereas the marker Bx215–148 was present (amplification product indicates repeat fruiting), indicating a closer association between repeat fruiting and the FANhyb19.1-derived markers than the Bx215-derived markers. More importantly, it was the absence of markers in this region that was more reliably predictive of once fruiting than the presence of the markers predictive of repeat fruiting.

### Predicting the expected segregation ratios of families

Family size, for the 31 families evaluated, ranged from 56 to 130 with an average size of 117 and only two families of fewer than 100 progenies (Table 2). For most families, the observed segregation could fit more than one closely related segregation ratio. Examples include 7:9 vs 3:5, 13:3 vs 3:1 vs 5:3, and 1:1 vs 7:9. In these families, genetic hypotheses derived from all probable segregation ratios were considered valid. Twenty-four selections were made from the 2016 repeat-fruiting seedling field. Thirteen survived and produced runners used to propagate plants for observation plants the following year. As with first-year-flowering breeding selections made in previous years, all thirteen selections made in 2016 flowered repeatedly through the 2017 summer and subsequent summers.

Based on the single-locus model, where the dominant allele confers repeat fruiting and also is expressed as flowering and fruiting in the first year, families from two once-fruiting parents would be expected to contain no individuals that would flower in their first year. Yet, only one family from two once-fruiting parents (B2472 × B1031) had no individuals flowering the first year, and both parents had a repeat-fruiting marker profile. Families from a repeat-fruiting parent and a once-fruiting parent were predicted to either contain half first-year-flowering progeny (if the repeat-fruiting parent was heterozygous) or all first-year-flowering progeny (if the repeat-fruiting parent was homozygous dominant). Eighteen families were derived from a cross between a repeat-fruiting parent and a once-fruiting parent, but only seven of these contained half first-year-flowering progeny. No families, regardless of phenotype or marker profile, contained only first-year-flowering progeny. Families from two repeat-fruiting parents were predicted to contain all first-year-flowering progeny (if one or both parents was homozygous dominant), or three times the number of first-year-flowering progeny as not flowering (if both parents were heterozygous). Just as none of the families contained all first-year-flowering progeny, none of the families with three times the number of first-year-flowering progeny were derived from two repeat-fruiting parents. One of the possible predicted segregation ratios based on known parental phenotype was the same as the observed segregation ratios for only eight out of thirty-one breeding families (Table 2). Therefore, the single-locus theory was generally not supported by observed segregation ratios as compared with ratios predicted by the parental phenotypes.

When the family segregation ratios were predicted by marker profiles, using the single-locus model, and a marker profile was conflicted for a parent (markers on one side of the repeat-fruiting locus indicated the parent was repeat fruiting, and markers on the other side indicated the parent was once-fruiting as in Additional file 3: Table S3), both options were considered for that parent (Table 2). Families from a parent with a conflicted marker profile and a parent with a once-fruiting marker profile were predicted to have progenies that either all flowered the first year, none flowered the first year, or half each (Table 2). Families from a parent with a conflicted marker profile and a parent with a repeat-fruiting marker profile were predicted to have either all first-year-flowering progeny, half first-year-flowering progeny, or three times the number of first-year-flowering progeny as not (Table 2). For only seven of the thirty-one families was the observed segregation ratio the same as one of the possible predicted segregation ratios, based on parental marker profiles (Table 2, Additional file 3: Table S3). When both the observed phenotypes and the marker profiles were used together to predict segregation ratios, only four families out of thirty-one segregated in one of the predicted ratios (Table 2). In addition, these four families were derived from at least one parent with a conflicted marker profile at the mapped locus (Table 2, Additional file 3: Table S3), reducing confidence in any explanation.

### Predicting the number of additional loci involved

The model that a single locus with a dominant allele confers repeat fruiting was not supported by the observed segregation ratios or the comparison between parental phenotypes and marker profiles at the mapped locus. A hypothesis that environmental conditions, such as high temperatures in Maryland, caused an individual with a repeat-fruiting marker profile to have a once-fruiting phenotype, would require that all individuals with a repeat-fruiting marker profile consistently have a once-fruiting phenotype in Maryland heat, where only about half did. A hypothesis that the mapped dominant repeat-fruiting allele had only 50% penetrance in Maryland’s heat could explain the disagreement between breeding-parent marker profiles and phenotypes, but would not allow the trait to have been genetically mapped. Incomplete penetrance of 50% would appear in mapping as completely random association of the trait and locus. By definition, the locus and phenotype would appear unlinked. That hypothesis would conflict with the two studies that mapped this locus in two populations in Maryland [9, 17, 18]. Similarly, an environmental explanation for an individual with a repeat-fruiting marker profile to have a once-fruiting phenotype half the time is not supported by several years of testing the parents in this study and observing consistent phenotypes across years. The simplest situation that would explain the disagreement is the presence of an additional locus not segregating, or at least not detected, in the mapping populations but segregating in some of the breeding populations. While the marker profiles of once-fruiting parents were in agreement with their phenotypes, many of the breeding parents with a repeat-fruiting marker profile had once-fruiting phenotypes, indicating something was interfering with and suppressing the mapped dominant repeat-fruiting gene. So the effect of an additional locus with non-additive suppressive effects was tested.

Hypothetical segregation ratios were calculated for all phenotypic and marker-profile combinations possible from the mapped locus combined with a dominant suppressor locus (81 parental combinations) or a recessive suppressor locus (81 combinations) (Table 3, Additional file 4: Table S4 and Additional file 5: Table S5). Two segregation ratios specific to a dominant suppressor epistatic on the mapped locus were 13 not flowering to 3 flowering, and 7 not flowering to 1 flowering. Two segregation ratios specific to a recessive suppressor epistatic on the mapped locus were 3 not flowering to 5 flowering, and 7 not flowering to 9 flowering.

**Table 3:** Possible segregation ratios of progeny resulting from crosses of repeat-fruiting (RB) and once-fruiting (jb) commercial strawberry parents. Possible progeny ratios are listed on the left. Parental combinations that would result in each segregation ratio are provided for the known mapped dominant gene conferring repeat fruiting with no suppressor, with a hypothetical dominant suppressor, or with a recessive suppressor of the known dominant gene

Progeny ratios	No suppressor			Dominant suppressor			Recessive suppressor		
All RB	RBxRB	RB and jb		RBxRB	RB and jb		RBxRB	RB and jb	jbxjb
1jb:3RB				RBxRB			RBxRB	RB and jb	
3jb:5RB							RBxRB	RB and jb	
7jb:9RB							RBxRB	RB and jb	
1jb:1RB		RB and jb			RB and jb	jbxjb		RB and jb	jbxjb
5jb:3RB					RB and jb			RB and jb	
3jb:1RB	RBxRB				RB and jb	jbxjb		RB and jb	jbxjb
13jb:3RB						jbxjb			
7jb:1RB						jbxjb			
All jb			jbxjb	RBxRB	RB and jb	jbxjb			jbxjb

Two families (Table 2) had segregation ratios that best fit those specific to a dominant epistatic suppressor acting on the mapped locus (Table 1). The families of 104 and 120 seedlings from B2475 × B1031 and B2488 × B1806 had a segregation ratios best fitting a ratio of 7:1 not-flowering to flowering (*p* > χ^2^ = 0.2356 and 0.1675, respectively). The next best fitting ratios (*p* > χ^2^ = 0.0415 and 0.035, respective to the two families) included 13:3, also indicative of a dominant suppressor, and 3:1, which could be explained by either a dominant or recessive suppressor with both parents being once fruiting (Additional file 4: Table S4 and Additional file 5: Table S5), but not by the single mapped locus.

Two families (Table 2) had segregation ratios that best fit those specific to a recessive epistatic suppressor acting on the mapped locus. The family of 104 seedlings from B1776RB × B1031 had a segregation ratio that best fit a 3:5 not-flowering to flowering (*p* > χ^2^ = 0.4178), and a 7:9 ratio (*p* > χ^2^ = 0.0379), and both are consistent with a cross between repeat-fruiting and once-fruiting parents (Additional file 4: Table S4 and Additional file 5: Table S5). The observed ratio also fit a 1:3 ratio (*p* > χ^2^ = 0.0415) which, in a cross between a once-fruiting and repeat-fruiting parents, can be explained by a recessive, but not a dominant, suppressor (Additional file 4: Table S4 and Additional file 5: Table S5) and could not be explained by only the mapped locus.

The family of 104 seedlings from B2396 × B1031 had a segregation ratio that best fit a ratio of 3:5 not-flowering to flowering (*p* > χ^2^ = 0.5434), followed by 7:9 (*p* > χ^2^ = 0.4890). Unfortunately, under the model of a recessive suppressor acting on a dominant mapped locus, those ratios are not expected if both parents are once-fruiting. The observed segregation ratio weakly fit a 1:1 ratio (*p* > χ^2^ = 0.0499), and can be explained by either a dominant or recessive suppressor (Additional file 4: Table S4 and Additional file 5: Table S5) but could not be explained by only the mapped locus, because both parents were once-fruiting.

The 15:1 segregation ratio observed for the family derived from B2480 and B1820, two parents with a once-fruiting phenotype and marker profile (Table 2, Additional file 5: Table S5), was not predicted by either a single dominant or recessive suppressor locus epistatic on the mapped locus. When both the parental phenotype and the marker profile for both parents were considered, the segregation ratios observed for several families could not be explained by a single suppressor locus, whether dominant or recessive.

Although many of the observed ratios could be explained by one model or the other, when multiple families with a parent in common were compared, there was no predicted genotype for the common parent that would lead to all the observed segregation ratios. These results indicate two or more suppressor loci acting on the mapped locus may exist in the Beltsville breeding program pool. That repeat fruiting was controlled by multiple loci, and influenced by environment, was proposed previously when a single mapping population was clonally propagated and evaluated in multiple locations [9, 16]. Differences between marker profile and phenotype for about half the parents in the current study suggest that the mapped locus is not the one affected by heat. Rather, one or more of the suppressor loci implicated in the seedling populations of the current study may be activated by heat.

With the model that repeat-fruiting is controlled by a dominant allele at only one locus, crosses between a once-fruiting parent and a repeat-fruiting parent should produce progeny that are all repeat fruiting, if the repeat fruiting parent is homozygous, or half repeat fruiting and half once-fruiting, if the repeat-fruiting parent is heterozygous. Thirty-six breeding families were planted from seedlings in a field in 2008 and evaluated during the summers of 2008 and 2009 (Additional file 6: Table S6). Twenty-eight families resulted from crosses between once-fruiting and repeat-fruiting parents. Two of these families contained all repeat-fruiting progeny in 2009, which is possible if repeat fruiting is controlled by a dominant allele only at the mapped locus. Yet, in 2008, the year the seedlings were planted and first evaluated, the progeny segregated and fit ratios that could be explained by an epistatic suppressor, either dominant or recessive (Additional file 4: Table S4, Additional file 5: Table S5 and Additional file 6: Table S6). Nineteen of these families segregated in a 1:1 ratio in 2009, which also is possible if repeat fruiting is controlled by a dominant allele only at the mapped locus. The segregation ratios in 2008, the year of planting, for 14 of these 19 families fit ratios that could be explained by an epistatic suppressor, either dominant or recessive (Additional file 4: Table S4, Additional file 5: Table S5 and Additional file 6: Table S6). Eight of the families planted in 2008 were derived from two once-fruiting parents. All of the progeny from three of these families were repeat fruiting in 2009, the second year of evaluation. In 2008, the first year of evaluation, the segregation ratio of all three families best fit a 7:1 ratio, once-fruiting to repeat-fruiting. The 7:1 ratio is only from a cross of two once-fruiting parents and only from a dominant suppressor acting epistatically on the mapped locus. This suppressor acts only in the first evaluation year, and not after the plants have been through a winter.

## Discussion

A mapping project usually identifies a few markers linked closely enough to a trait of interest to be potentially useful in breeding, especially if the mapping project states which markers are in coupling and which are in repulsion with the trait. However, since breeding programs often use multiple parents of diverse backgrounds, the closest markers identified in a mapping population may not be useful with some breeding parents. It is helpful, therefore, to have available multiple markers linked to the trait for use in manipulating a trait in breeding populations. In this study, the availability of the strawberry genomic sequence allowed us to search for additional markers near the trait of interest. This process of mining large sequence-scaffolds of cultivated strawberry, through sequence similarity to primer sequences previously mapped to the trait, was successful and has been used in other studies [24]. Five new SSR primer pairs were developed and mapped to repeat fruiting in one of the populations tested, and four mapped to repeat fruiting in the other population tested. The success of this process highlights the value of continued work of assembling and annotating whole-genome sequences, though it is often a tedious and thankless job.

Characterizing breeding parents with 19 markers, instead of just one or two, provided initial confidence that disagreements between marker profile and known phenotype were not due to a simple recombination event between the gene conferring the trait and the closest marker. Markers most strongly associated with repeat fruiting using the Kruskal-Wallis nonparametric test were not in complete agreement with the maps generated by JoinMap, and the maps were not in complete agreement with each other. The Kruskal-Wallis test was helpful, as a follow-up to linkage mapping, in identifying which markers are most likely to predict the phenotype, either in identifying desired seedlings, or in choosing parents. The combination of mapping and the Kruskal-Wallis test in two populations was helpful in identifying the three markers (FANhyb19.1–15-121 and FANhyb19.1–4-189, and Bx215–148) that best predict that a parent with a once-fruiting marker profile (where these three markers were absent would also have a once-fruiting phenotype. All three were linked in coupling to the dominant repeat-fruiting locus. Two of these markers (FANhyb19.1–15-121 and FANhyb19.1–4-189) were developed in this study from a single contiguous sequence and mapped 1.9 cM from repeat fruiting in one mapping population and 8.4 cM and 10.1 cM from repeat fruiting in the other population. Mapping differences were not considered to be evidence of anything more complicated than simple experimental error.

Although the absence of these three markers was very useful in identifying once-fruiting parents, their presence could not be used to identify repeat-fruiting parents and incorrectly predicted repeat fruiting in about half the parents. When FANhyb19.1–15-121 and FANhyb19.1–4-189 were present or Bx215–148 was present, there was about a 43% chance of the genotype being repeat fruiting. Bx215–128 was only 85% positively predictive in a collection of germplasm [29]. And the presence of both Bx083_206 and Bx215_131, 7.3 cM and 1108 Kb apart, and hypothesized to flank the gene controlling repeat fruiting, predicted repeat fruiting in only 82% (14 of 17 independent genotypes with a repeat fruiting phenotype) [24]. Double recombination between the markers was suggested to be responsible [24]. Only one genomic region was detected in multiple mapping studies, and the phenotypes of many tested cultivars were well known in many environments, suggesting discrepancies between marker profiles and phenotypes were due to the action of multiple genes strongly affected by environment [25]. These independent findings suggest the existence of at least one, and probably more, additional independent loci with epistatic effects on the one mapped. Because the recessive once-fruiting phenotype is reliably predicted by the absence of these same markers, this indicates that the hypothetical epistatic loci act as suppressor of the mapped dominant repeat-fruiting locus. And it is the unmapped epistatic loci, rather than the mapped locus, that are most likely to be affected by environmental effects and/or incomplete penetrance [9, 25].

Marker profiles combined with known phenotypes of the parents failed to predict observed segregation ratios for the progeny of all but four parental combinations. These two failures of the hypothesis that repeat fruiting was entirely controlled by one locus with a completely dominant allele conferring repeat fruiting lead to the supposition that at least one additional and independent locus affecting repeat fruiting exists in this breeding program pool. Because only one locus was detected through mapping processes generally assuming additivity of genetic effects, the hypothetical additional locus was presumed to have non-additive genetic effects. Epistasis was considered more likely than the non-additive effect of incomplete penetrance, because of the combined evidence acquired through genetic linkage and breeding-parent phenotype and marker profile comparison. Where one line of evidence allowed multiple explanations, the combined evidence did not, and ruled out incomplete penetrance of the mapped locus as a significant reason for discrepancies observed. This evidence also implied that the second locus acted as a suppressor of repeat fruiting through epistasis on the mapped locus.

Hypothetical segregation ratios were calculated for all phenotypic and marker-profile combinations possible from the mapped locus combined with a dominant suppressor locus (81 parental combinations) or a recessive suppressor locus (81 combinations) (Additional file 4: Table S4 and Additional file 5: Table S5). Not all of the observed ratios could be explained by one model or the other, and when multiple families with a common parent were compared, there was no predicted genotype for the common parent that would lead to all of the observed segregation ratios. The segregation ratios of some of the families provided strong evidence of additional loci, some dominant, some recessive, and perhaps some acting together, to affect repeat fruiting as conferred by the dominant allele of the single mapped locus.

Previously, two markers flanking the repeat-fruiting region were used to search for sequence identity in the diploid reference genome v2.0.a1 sequence and found to span a 1108 Kb region containing 234 predicted genes, including 15 classified as flowering-associated, one of which had sequence homology to *FLOWERING LOCUS T* (FLT) [24], a key to floral regulation in *F. vesca* [30]. The action of *FLOWERING LOCUS T* is strongly affected by *CONSTANS*, a well-known example of epistasis on a molecular level [31]. In *F. vesca*, overexpression of a homolog to *SUPPRESSOR OF OVEREXPRESSION OF CONSTANS1* (Fv SOC1) repressed flower initiation under inductive short days, whereas its silencing caused continuous flowering in both short days and noninductive long days, similar to mutants in the floral repressor *F. vesca TERMINAL FLOWER1* (Fv TFL1), and that Fv SOC1 activates Fv TFL1 in the shoot apex, leading to the repression of flowering in strawberry [32]. Just considering how SOC1 acts on CONSTANS, which acts on FLT, could support the concept of a single locus affected epistatically by two suppressor loci.

Flowering is also strongly affected by vernalization epistatically, and a vernalization requirement in some strawberry genotypes, but not others, has been reported [33]. It’s possible that the dominant epistatic suppressor that only seems to act the first year of evaluation and not after experiencing a winter is a gene affecting vernalization, consistent with the idea that repeat-fruiting is affected by environment, and possibly incomplete penetrance.

Sixty-six gene homologs that, by sequence similarity, putatively correspond to genes of all known genetic flowering pathways have been identified in diploid *F. vesca* [34]. These are just a few examples supporting the idea that other loci could affect the action of the mapped repeat-fruiting locus on HG IV. The cumulative knowledge of the number of genes affecting flowering in plants and their known epistatic interactions with each other raises questions as to why only one locus has thus far been mapped controlling repeat fruiting in strawberry.

It is likely that only one locus was detected because of the methods used to phenotype and the populations used. Data were collected from the ‘Tribute’ × ‘Honeoye’ after the seedling year so the population could be clonally propagated and tested in multiple states [9]. When the cross was repeated, and tested in Maryland, 37 of the seedlings of 124 flowered and fruited through the summer of the year of planting, best fitting a segregation ratio of 3 not-flowering to 1 flowering. In the following summer, 69 of the surviving 122 progeny flowered and fruited through the summer, best fitting a 1:1 ratio. As with many families evaluated in the Beltsville breeding program in 2008 and 2009, several repeat-fruiting progeny were suppressed from flowering in the year of planting (Additional file 6: Table S6), but not in the years afterwards, when data were collected for mapping [9, 16]. Perhaps this is a missed opportunity to map a vernalization gene in strawberry.

Mapping populations often are developed from parents with extreme opposing phenotypes. Both the ‘Tribute’ × ‘Honeoye’ and ‘Delmarvel’ × ‘Selva’ populations were used because the parents had opposing phenotypes. The current study showed that segregating families can be obtained from two parents with repeat-fruiting marker profiles and repeat-fruiting phenotypes (Table 2) or from two once-fruiting parents (Additional file 6: Table S6). Mapping populations often are selected that allow qualitative evaluation and discovery of only one locus at a time, as with the ‘Delmarvel’ × ‘Selva population, which segregated 1:1.

Mapping software generally assumes additive genetic effects, including some of the newer approaches being used [25, 35]. Some mapping programs allow the user to mask the effect of one locus in order to identify other loci. Loci with epistatic effects on grape proanthocyanidin composition were identified using R/QTL [36] which has several requirements our populations did not meet. The multiple QTL method (MQM), available with the MapQTL software [37] used in this study, uses the most significantly linked markers to detected QTL as cofactors to control the genetic background and allow detection of additional significant QTL. This approach did not detect additional significant QTL in either the ‘Delmarvel’ × ‘Selva’ population or the ‘Tribute’ × ‘Honeoye’ population [17, 18].

Again, it is likely that only one locus was detected by multiple research groups with multiple populations because of how/when the phenotypic data were collected and the genetic composition of the parents of the populations. Yet, this does not explain the unlikely finding of the same locus by these multiple groups. If the multiple loci hypothesized to control repeat fruiting are independent in both genetic distance and expression, at least one of the groups should have found a unique locus affecting repeat fruiting. The fact that all groups found the same locus indicates that the mapped locus is the only locus capable of influencing segregation independently of the others. The loci that have not yet been mapped are dependent on the mapped locus to be revealed. Whether the parents have once-fruiting or repeat-fruiting phenotypes, the epistatic suppressor loci can be mapped most efficiently when both parents have the repeat-fruiting marker profile for the locus already mapped. Possible examples from the current study would include any of the repeat-fruiting parents B2409RB, B2325, or B2411RB crossed with the once-fruiting parent B1806, which has the repeat-fruiting marker profile, as the resulting families segregated in a ratio close to 1:1 (Table 2). A breeding program that characterizes all breeding parents molecularly at known loci, in addition to phenotypically, could be helpful identifying the populations needed for the sequential mapping efforts from annual breeding families.

## Conclusions

Searching genomic sequence scaffolds with sequences from primers genetically linked to repeat fruiting yielded five new markers linked closely to repeat fruiting. The approach may not be very efficient, as most of the amplification products were not useful as planned and either did not map or mapped to a non-target linkage group. Additional effort to annotate genomic sequences would be helpful to this and other genetic study types.

Similar to what was observed by others [24, 25, 29, 35], a once-fruiting parental marker profile was nearly always indicative of a once-fruiting phenotype, yet a repeat-fruiting parental marker profile falsely predicted a repeat-fruiting phenotype, about half the time in the current study. The cause of this discrepancy cannot be incomplete penetrance of the dominant allele. Penetrance of 50% would have resulted in random association between the trait and the genomic region around the causative gene, preventing the mapping of the trait by several research groups [9, 16–18, 23–25], as genetic mapping requires non-random association between traits and markers. The simplest explanation is that one or more independent loci are present which suppress repeat-fruiting in individuals with a repeat-fruiting marker profile but a once-fruiting phenotype. This is an example of epistasis.

The existence of one or more epistatic suppressor loci was confirmed by the segregation of progeny from parents characterized by marker profile and multiple years of phenotypic evaluation. Segregation ratios specific to an epistatic suppressor acting on the mapped locus were observed in four families. The segregation ratios for two families were best explained by a dominant suppressor acting on the mapped locus, and, for the other two, by a recessive suppressor.

Although incomplete penetrance may not have significant direct effect on the mapped locus, it and the growing environment may be a significant factor acting on epistatic suppressor loci. This may explain the phenotypic discrepancies for individuals grown in different states as noted generally in the strawberry industry [25] and genetic linkage studies [9, 16]. Environment and/or incomplete penetrance effects on the epistatic suppressor loci also could contribute to the slightly different maps generated in the current study (Fig. 1).

One of the suppressor loci acting epistatically on the mapped locus is a dominant suppressor that acts only in the first year, the year new seedlings are planted, but not in subsequent years.

## Methods

### Developing new SSR markers for the repeat-fruiting region

To further saturate the repeat fruiting region with markers for this study, additional SSR markers were designed using markers previously mapped to the region [17, 18]. The forward and reverse primer sequences of each SSR marker linked to repeat fruiting (Table 1) were used to search for similar sequences in the reference genome (FANhybrid_r1.2) of the cultivated strawberry, *F.* ×*ananassa* [38], from the Strawberry GARDEN website (http://strawberry-garden.kazusa.or.jp, accessed April 2016). Custom scripts (available upon request) using Python programming language were used that allowed a mismatch of up to 4 bp (approximate exact-matching algorithm). Genome-sequence scaffolds containing regions with high sequence similarity to the primer sequences were selected for designing new SSR markers for the repeat-fruiting region. Repetitive motifs were identified by SSR identification tool (SSRIT, http://www.gramene.org/db/markers/ssrtool). Oligonucleotide primers were designed in the SSR-flanking sequences using Genious 7.2 software tool [39]. Primers were designed to meet the following parameters: amplicon size between 100 and 450 bp; primer length from 18 to 25 bp, GC content between 40 and 60%; melting temperature between 55 °C and 65 °C (59 °C optimum).

The SSR primer pairs developed were tested for polymorphism detection with the mapping parents (‘Tribute’ and Honeoye’, ‘Delmarvel’ and ‘Selva’). The populations derived from them were genotyped with those primers that detected polymorphisms. Details regarding these two mapping populations were described previously [17, 18]. The mapping populations were genotyped with eight additional SSR markers (Bx052, Bx056, Bx059, Bx064, Bx083, Bx089, Bx215, Bx250) linked to repeat fruiting [24].

For use in parental screens and genetic mapping, the 5′ ends of the forward primers were modified with the addition of the M13 sequence (5′-TGTAAAACGACGGCCAGT-3′), and separate M13 primers were labeled with the fluorescent dyes 6-FAM, VIC, NED and PET (Applied Biosystems, Foster City, CA, USA) [40]. The 5′ ends of the reverse primers were modified by the addition of a short sequence (GT, GTT or GTTT) to facilitate adenylation for more uniform product sizes [41]. Polymerase chain reaction (PCR) amplifications were performed as described [40]. Primer pairs were first tested with the mapping parents, and those that generated amplicons polymorphic between parents were combined by product size and fluorescent dye color into multiplexes of up to four primer-pair products after amplification in the mapping population. Samples for electrophoresis were prepared as described [17]. PCR products were separated by capillary electrophoresis with an ABI3730 DNA Analyzer (Applied Biosystems Inc., Foster City, CA, USA). Allele sizes were determined using GeneMapper 3.5 software (Applied Biosystem) and GENSCAN 500HD (LIZ) as an internal size standard. To map the SSRs in ‘Tribute’ × ‘Honeoye’ and ‘Delmarvel’ × ‘Selva’ populations, linkage analyses and genetic linkage map construction were performed as described [17, 18]. The non-parametric method of Kruskal-Walis testing [42] was used to identify the markers most tightly linked to the repeat-fruiting region. Markers with a *P* ≤ 0.005 were selected as the most tightly linked.

### Growing repeat-fruiting seedlings

Seeds were germinated and transplanted to 72-well trays in a greenhouse. Seedlings were planted on the Henry A. Wallace Beltsville Agricultural Research Center Linkage Farm (39°01′22.43″N, 76°54′59.26″W, 43.6 m elevation). Observation plots and replicated evaluation plots were on the North Farm (39°01′48.42″N, 76°56′07.99″W, 49.4 m elevation). Daylength was recorded at Greenbelt, Maryland. Air temperatures (2016) were recorded at 1.5 m high at the Linkage Farm. A 2-m high weather station located less than 300 m from all fields on the Linkage Farm measured sheltered temperatures with a CS107 temperature sensor inside a 41,303-5A RM Young six-plate solar radiation shield (Campbell Scientific, Logan, UT). Measurements were taken and collected every 10 s, and averaged at 15-min intervals. Data were transmitted through a 900 Mhz spread-spectrum protocol using an RF450 radio (Campbell Scientific) to a NL100 network link (Campbell Scientific) for display with Vista Data Vision sensor data management software, (Reykjavik, Iceland).

An annual plasticulture production system [43], used to grow and select once-fruiting strawberries at Beltsville, was adapted in 2008 for the purpose of evaluating seedlings expected to segregate for repeat fruiting, and to maximize flowering and fruit quality through hot summer conditions. Raised beds were made in the field with a Model 2600 Series II Plastic Mulch Layer (Rain-Flo Irrigation, East Earl, PA) modified to form a bed with a 7-cm crown to help shed rain water. The beds were covered with white-on-black plastic mulch to reduce soil temperature compared with black plastic mulch, as soil temperature was key to summer fruit production from repeat-fruiting strawberries [10]. Two lines of 15 mil drip tape (30 cm in-line spacing) were installed the length of each bed as the bed was made. They were placed 7 cm below the bed surface and 16 cm to each side of bed center for maximum availability to the seedlings that were planted in two rows, 18 cm from bed center, the length of the bed. After planting, the seedlings were watered and fertilized weekly with 5.6 kg·ha^− 1^ nitrogen. Small weeds were regularly removed from planting holes by hand. Strawberry seedling stolons or “runners” were pulled from under the plastic mulch, away from neighboring seedlings, and away from the bed center to prevent confounding of phenotypes in neighboring planting holes.

### Identifying “repeat-fruiting” genotypes in their seedling year

Previous studies described how repeat-fruiting genotypes could be identified in their seedling year. Seeds were germinated in late winter to early spring, established in the field in the spring, and evaluated for flowering and fruiting from mid-summer to early fall [12]. Alternatively, flowering in the first six weeks of the first year was found to indicate that the selection would be repeat fruiting [13]. At Beltsville, from 2008 to 2011, several seedling families expected to segregate for repeat fruiting were evaluated for flowering in their seedling year and repeat flowering/fruiting in their second year. A total of 67 families, ranging in size up to 120 progeny per family, were planted in 2008, 48 families were planted in 2009, 46 families in 2010, and 24 families in 2011. Seedlings were numbered, and data were recorded (flowering/not-flowering) for every individual seedling every two weeks from late June through early October. In addition, the first time a seedling flowered, it was marked with a plastic flag on a meter-high wire staff. The field was maintained through the winter and cultivated to encourage fruiting the following year. In June each year, after the first spring fruiting period ended for all fields in the program, flowering data were again collected for the seedlings planted the previous year. In this way, it could be determined if seedlings that flowered the first year would fruit repeatedly the second year after spring fruiting was finished. In these four years, many seedlings that did not flower in the first year did flower repeatedly the second summer (Additional file 6: Table S6), and in subsequent years when selected as potential cultivars. All seedlings that flowered the first year, and were healthy the second year, flowered and fruited repeatedly through the second summer. After 2011, seedlings that flowered the year of planting were simply marked with a wire, and the number of flowering seedlings was counted as a portion of the total number of live plants in each family; segregation data were not collected the following year.

### Comparing breeding parent phenotypes and marker profiles

**Fig. 2:**
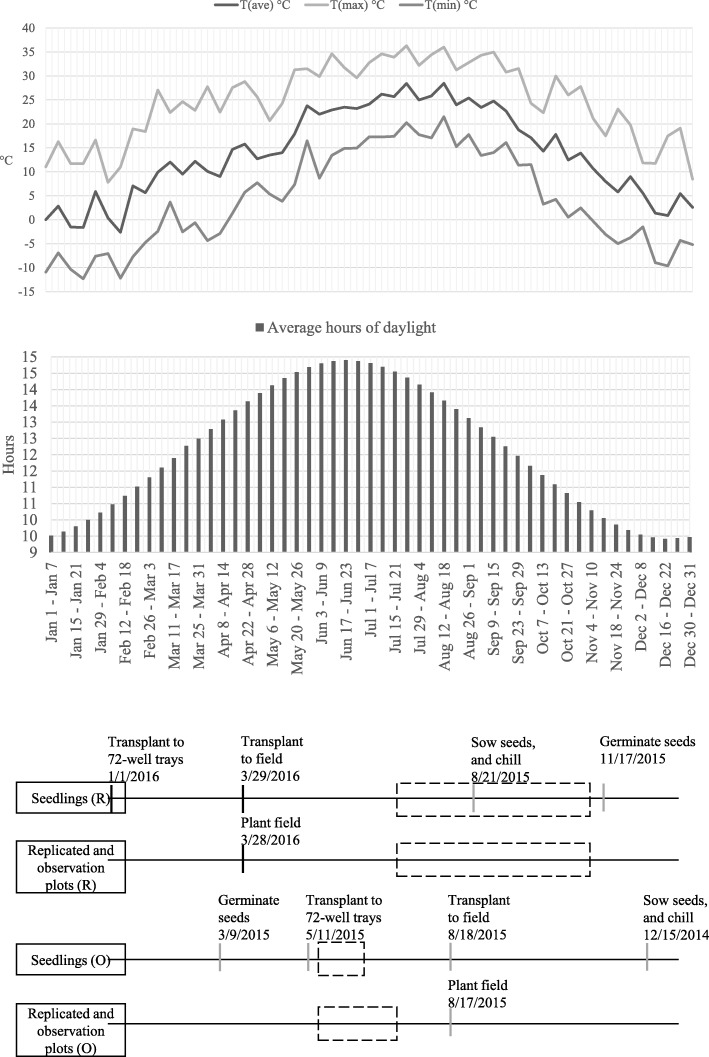
Progression of annual activities for the USDA-ARS strawberry breeding program for once-fruiting (O) and repeat-fruiting (R) strawberry genotypes. Activities from seed germination to plant evaluation are expressed on horizontal time lines associated with temperatures and daylength for the repeat-fruiting seedling evaluation year (2016). A vertical bar marks the starting date for an activity that occurred the same year (black) or years prior (grey) to the evaluation year (2016). A black dashed-line box represents 2016 flower/fruit-evaluation periods. Seeds were germinated and transplanted to 72-well trays in a greenhouse. Seedlings were planted on the Henry A. Wallace Beltsville Agricultural Research Center Linkage Farm (39°01′22.43″N, 76°54′59.26″W, 43.6 m elevation). Observation plots and replicated evaluation plots were on the North Farm (39°01′48.42″N, 76°56′07.99″W, 49.4 m elevation). Daylength was recorded at Greenbelt, Maryland. Air temperatures (2016) were recorded at 1.5 m high at the Linkage Farm

Since 2007, annual crossing plans were divided into two types of crosses: those designed to be all once fruiting (“once-fruiting” crosses), planted in July on black plastic beds and fruited the following year; and those designed to segregate for repeat fruiting (“repeat-fruiting” crosses), planted in March in white-plastic beds and fruited the same year (Fig. 2). Seedlings from either type were identified annually as having potential to be cultivars, referred to as “selections”, and were assigned selection numbers that identified them through additional evaluation in multiple years. Selections were evaluated in six-plant “observation plots” the year after selection as a seedling. Selections from once-fruiting crosses were propagated by runners in a greenhouse and planted the August after selection, a year after the seedling was planted, but the same year it was selected. These selections were subjectively evaluated the following spring, and if selected again, were propagated and evaluated again in replicated plots [44]. All selections in observation plots and replicated plots were evaluated after the main fruiting period was ended to identify any that might flower again, even though they were not selected as such. Since once-fruiting and repeat-fruiting crosses were first planted in separate fields at different times of year in 2008, no seedling selected from the once-fruiting seedling field has since been re-classified as repeat fruiting.

Selections from repeat-fruiting crosses were propagated by runners in a greenhouse then provided ten to twelve weeks chilling in a walk-in cooler before planting in observation plots the next spring, less than a year after they were selected. These selections were evaluated the same year they were planted in the observation plots, a year after they were evaluated and selected as seedlings. Selections that failed to make runners were not evaluated further. Repeat-fruiting selections were checked multiple times in observation plots to make sure they were still fruiting all summer and fall and to observe how fruit quality changed through the year. Selections not discarded at this step in the breeding process were used annually as parents in the breeding program.

Selections that were used as parents in 2015 to generate families segregating for repeat fruiting in 2016 were characterized with all the primer pairs that mapped to the entire repeat-fruiting linkage group. Deoxyribonucleic acid (DNA) was collected from all breeding selections in the strawberry breeding program for characterization with molecular markers of interest. Each year, from each observation plot, young leaves, still folded, were collected and desiccated for long-term storage. The leaves from an observation plot were collected and placed in a labeled glassine envelope (Clearbags, Selmer, TN). Up to six envelopes, representing one selection each, were placed in a gallon-size sealable plastic bag with around 177 mL molecular sieve desiccant, Type 4A round beads, form 8 × 12 (ADCOA, Gardena, CA), with a humidity indicator card. Air was left in the sealed bag to facilitate drying, and the bag was turned repeatedly in the first two days until the indicator card stayed blue. The desiccant was refreshed annually. From the parents of the strawberry breeding project’s 2016 repeat-fruiting seedling families, dry leaf tissue was fragmented using a TissueLyser (Qiagen, Inc.), and DNA was extracted using the DNeasy® Plant Maxi Kit (Qiagen, Inc.) in a QIAcube® machine (Qiagen, Inc.).

PCRs were conducted with the breeding parents as described for mapping the new markers. PCRs were completed in 96-well plates with two reactions per selection as replication, and one primer pair per plate. Mapping parents, ‘Tribute’, ‘Honeoye’, ‘Delmarvel’, and ‘Selva’, were included on each plate to confirm product size.

### Predicting the expected segregation ratios of families

Segregation ratios for flowering in the first year (repeat fruiting in subsequent years) were predicted for each breeding family resulting from the breeding parents. Predictions were based on both the known field phenotypes of the parents (over multiple years) and the molecular profile of the parents of each family. Predictions also were based on the results from multiple studies indicating repeat fruiting was conferred by a dominant allele at a single locus [18, 23–25]. In this model, once-fruiting parents were homozygous, and could pass only the once-fruiting allele. Repeat-fruiting parents could have been either homozygous dominant and pass only the repeat-fruiting allele, or heterozygous and could pass either allele. For parents with marker data not in agreement along the part of the linkage group most closely associated with repeat fruiting (Additional file 3: Table S3), both options were considered in making segregation predictions (Table 2). When other segregation ratios needed to be tested, they were based on the assumption of the presence of the known single locus (with a dominant repeat-fruiting allele), combined with other independent loci, with one allele conferring one or the other flowering pattern and having an epistatic suppressive effect (Additional file 4: Table S4 and Additional file 5: Table S5).

### Determining the actual segregation ratios for each seedling family

The strawberry breeding project’s seedling families expected to segregate for repeat fruiting were planted in a field at the Beltsville Agricultural Research Center (BARC) in Spring 2016. Throughout the summer, from late June through late September, any plant that flowered was marked with a white flag. Selections were made from among these seedlings and evaluated in observation plots the following year. For each family, the number of plants that flowered, the number of plants that did not flower, and the number of diseased or dead plants were recorded. Observed segregation ratios were compared with expected segregation ratios using the Chi-square goodness-of-fit test. The probability of a greater Chi-square value was calculated rather than using an exclusion probability.

## Additional files

Additional file 1: **Table S1.** List of commercial strawberry genome sequence scaffolds selected for primer design and characteristics of the new SSR primer pairs developed. (DOCX 27 kb)

Additional file 2: **Table S2.** Amplification products from SSR markers in ‘Delmarvel’ × ‘Selva’ and ‘Tribute’ × ‘Honeoye’ strawberry (F. ×ananassa) F1 mapping populations (DOCX 24 kb)

Additional file 3: **Table S3.** Breeding-parent marker profiles using all markers of Linkage Group IV, which is associated with repeat-fruiting in commercial strawberry (*Fragaria* ×*ananassa*), that were amplified from the repeat-fruiting mapping parent and had a significant association with repeat fruiting when using the Kruskal-Wallis test. Markers are listed in order according to Kruskal-Wallis test value for mapping population generated by 'Tribute' × 'Honeoye'. Primer amplification of a product is designated by a "1", no amplification of the product when others were amplified by the primers is designated by a "0", and no amplification of any products from the primers was considered a failed reaction and designated by "m" for "missing". Marker products associated with once-fruiting are colored grey ("1" if the marker is in coupling, and "0" for the marker in repulsion). (XLSX 17 kb)

Additional file 4: **Table S4.** Calculations of segregation ratios for progeny families from all possible phenotype/genotype parental combinations for a two-locus model to explain repeat fruiting *vs* once-fruiting of strawberry. Calculations assume the primary, already mapped locus, has a dominant allele (RB) conferring repeat fruiting, and a recessive allele (jb) conferring once-fruiting. Repeat-fruiting parental and progeny phenotypes are highlighted in dull yellow, and once-fruiting phenotypes are highlighted in pale green. On the left of the grey vertical bar are calculations that assume the presence of a second locus with a completely dominant allele (STOP) conferring epistatic suppression of the mapped locus when homozygous (STOP STOP) or heterozygous (STOP stop), and a recessive allele (stop) that does not affect the mapped locus). On the right of the grey vertical bar are calculations that assume the presence of a second locus with a recessive allele (stop) conferring epistatic suppression of (or failure to support) the mapped locus when homozygous (stop stop), and a completely dominant allele (STOP) that does not change the phenotype conferred by the mapped locus. Horizontal grey and purple bars separate different genotypic parental-combination possibilities for similar phenotypic possibilities. Resulting progeny phenotypes are characterized as repeat-fruiting (RB) or once-fruiting (jb) similar to the mapped locus. Expected resulting segregation ratios are highlighted in bright yellow boxes. (XLSX 235 kb)

Additional file 5: **Table S5.** Segregation ratios for progeny families from all possible phenotype/genotype parental combinations for a two-locus model to explain repeat fruiting vs once-fruiting of strawberry. Segregation ratios assume the primary, already mapped locus, has a dominant allele (RB) conferring repeat fruiting, and a recessive allele (jb) conferring once-fruiting. Repeat-fruiting parental phenotypes are highlighted in dull yellow, and once-fruiting phenotypes are highlighted in pale green. On the left of the grey vertical bar are segregations ratios that assume the presence of a second locus with a completely dominant allele (STOP) conferring epistatic suppression of the mapped locus when homozygous (STOP STOP) or heterozygous (STOP stop), and a recessive allele (stop) that does not affect the mapped locus). On the right of the grey vertical bar are segregation ratios that assume the presence of a second locus with a recessive allele (stop) conferring epistatic suppression of (or failure to support) the mapped locus when homozygous (stop stop), and a completely dominant allele (STOP) that does not change the phenotype conferred by the mapped locus. Horizontal grey and purple bars separate different genotypic parental-combination possibilities for similar phenotypic possibilities. Resulting progeny phenotypes are characterized as repeat-fruiting (RB) or once-fruiting (jb) similar to the mapped locus. Similar segregation ratios are highlighted in similar colors, and simplified to the right if appropriate, with non-segregation designated as RB for all repeat-fruiting, and jb for all once-fruiting. (XLSX 41 kb)

Additional file 6: **Table S6.** Segregating strawberry breeding families planted 2008 and evaluated the summers of 2008 and 2009. Parental and progeny phenotypes were classified as once-fruiting (jb) or repeat fruiting (RB) for each summer. Segregation ratios were calculated and tested for Chi-square goodness of fit. Chi-test probabilities over 0.000001 were highlighted in light green. The best fitting ratio or ratios were determined for the year of planting and for the subsequent year. Differences in segregation ratio from one year to the next occurred for several families. (XLSX 58 kb)

## Data Availability

All data generated or analyzed during this study are included in this published article [and its supplementary information files].

## Abbreviations

BARC: Beltsville Agricultural Research Center
DN: Day neutral
DNA: Deoxyribonucleic acid
FLT: *FLOWERING LOCUS T*
Fv TFL1: *Fragaria vesca TERMINAL FLOWER1*
HG: Homoeologous group
LG: Linkage group
MQM: Multiple QTL method
PCR: Polymerase chain reactions
QTL: Quantitative-trait loci
SD: Short day
SOC1: *SUPPRESSOR OF OVEREXPRESSION OF CONSTANS1*
SSR: Simple-sequence repeat
SSRIT: SSR identification toolkit
US: United States
USD: United States Dollars
USDA-ARS: United States Department of Agriculture - Agricultural Research Service
