# Supplementary material for: Evidence of epistatic suppression of repeat fruiting in cultivated strawberry
Source: BMC Plant Biol. 2019 Sep 5;19:386. doi: 10.1186/s12870-019-1984-7 (PMC6729047; doi:10.1186/s12870-019-1984-7)
Supplement: Supplementary file 1 — Table S1. List of commercial strawberry genome sequence scaffolds selected for primer design and characteristics of the new SSR primer pairs developed. (DOCX 27 kb) [file 12870_2019_1984_MOESM1_ESM.docx]

Table S1 List of commercial strawberry genome sequence scaffolds selected for primer design and characteristics of the new SSR primer pairs developed

| Scaffold | SSR name | Forward primer (5’-> 3’) | Reverse primer (5’-> 3’) | SSR motif | Expected size (bp) | Position on scaffold (bp) |
| --- | --- | --- | --- | --- | --- | --- |
| FANhyb_rscf00000019.1 | FANhyb_19.1-17 | ATGGAAGCCCAACCTATAGTA | GCAGATCAATCTTCTTCTTCTC | (AACATG)4 | 102 | 98739-98841 |
|  | FANhyb_19.1-16 | GAAGTTGCACCGAACGTAGC | CAGGAGATCGCAAACCACCT | (AGA)4 | 141 | 99119-99260 |
|  | FANhyb_19.1-15 | TTCTCCATCTTCACATTTCTG | TGCTCTCTTTTGCTAAGTACG | (TTGGA)5 | 112 | 117514-117626 |
|  | FANhyb_19.1-14 | ATTCAGAGAATTCGGGTTTAG | AAGCTGTCTTCTTCTGAGTCC | (GAA)4 | 102 | 121110-121212 |
|  | FANhyb_19.1-13 | TATACTTATGGTTGGCCTCCT | CCAGGAGACCTAATGAACTTT | (GAAA)3 | 158 | 121571-121729 |
|  | FANhyb_19.1-2 | ACAACACACTGATGATGGAGTCT | TGGTCAATTTCGGTTGTCCC | (CTT)4 | 383 | 129507-129893 |
|  | FANhyb_19.1-1 | GCAACTTCTCAACTTTAGCAC | CGTGATGAGTTCAAATTAAGG | (CTT)4 | 232 | 129583-129815 |
|  | FANhyb_19.1-3 | CGCAGCACCAGTCTTTCATG | AACCCTCCTCCTCCTCCTTC | (AAG)5 | 402 | 135579-135981 |
|  | FANhyb_19.1-4 | GGTCTGGATTTAGGAGAGAAA | TCTTCTTCTTTCATCAGAAGC | (AGG)5 | 149 | 135913-136062 |
|  | FANhyb_19.1-5 | TGGTTGTGGTATTGGGTGGG | AGCCATTGGTGTGATCCGAA | (AGA)4 | 263 | 136005-136268 |
|  | FANhyb_19.1-7 | AGAGGATTGGGTGGTCAGGA | CCCACCCAATACCACAACCA | (AAG)4 | 143 | 138056-138199 |
|  | FANhyb_19.1-6 | GGTTTAGGAGAGAAACCAATC | GATTACACTCGCACACTTCTC | (AGG)4 | 154 | 138111-138265 |
|  | FANhyb_19.1-8 | ACGGTTTATCGGCTGGAGTG | AATGTTCTCGGAGGCATGCA | (GA)7 | 289 | 139825-140114 |
|  | FANhyb_19.1-9 | CATTGGGGTTTGATGATATAG | TGCACTACAGGAGCTTTTTAC | (GA)7 | 121 | 139942-140063 |
|  | FANhyb_19.1-10 | TAAAGTTTTCATTGCGATTG | ATGCTTGTGGTAACTATGCAC | (AATA)3 | 160 | 141429-141589 |
|  | FANhyb_19.1-12 | TCCTTGGCCATGGCATTCAT | TCCCTCATTGGAAGAGCCCT | (AT)8 | 273 | 147060-147333 |
|  | FANhyb_19.1-11 | ATGCAACTCCTTTACAAGAAA | GGTGTAATGCCATATCCCTAT | (AT)8 | 148 | 147225-147373 |
|  |  |  |  |  |  |  |
| FANhyb_rscf00000094.1 | FANhyb_94.1 -1 | GAACTTTTGTAACATGGCATC | CAGGGGTACAAGACACATAAG | (TTC)3 | 145 | 117051-117196 |
|  |  |  |  |  |  |  |
| FANhyb_rscf00000611.1 | FANhyb_611.1 -1 | TCCCACAATCATCGACCCAT | TCGGCTGCTTCTTGGTACAG | (CTC)4 | 267 | 495-762 |
|  | FANhyb_611.1 -2 | ACTGGACCTGCATTGTAGTAG | CAACATTCCCATTCTCAATAG | (TTGTAG)3 | 123 | 2058-2180 |
|  |  |  |  |  |  |  |
| FANhyb_rscf00000758.1 | FANhyb_758.1-4 | GTAGCCAAATCAACCAAAACT | CCTTTCCCTAAATGTTTTCTT | (AAC)4 | 110 | 20944-21054 |
|  | FANhyb_758.1-3 | TGCATACGTGGCTCATGAGT | AAAGGAAGACCGTGCGCATA | (ATTAA)3 | 399 | 34183-34582 |
|  | FANhyb_758.1-1 | CACCCATTAAGGATGAAAATA | AGGAATGTCCATTAAATGTT | (TA)7 | 142 | 38979-39121 |
|  | FANhyb_758.1-2 | GGAAAGGACTGTTTGACAATA | ACTGCTCCAATATGAGATTCC | (TGA)4 | 105 | 40055-40160 |
|  |  |  |  |  |  |  |
| FANhyb_rscf00000064.1 | FANhyb_64.1-4 | AAGAAGAAACAAAAACACACG | GCGAAGAAGAAGAAGAAGAAG | (CT)7 | 109 | 113819-113928 |
|  | FANhyb_64.1-3 | CCGCTATCTCTACGTATTTGA | TAGCATGTGTAAAGGAGGATG | (CAAC)3 | 160 | 119292-119452 |
|  | FANhyb_64.1-2 | TCTCAATACAGAAGAGCTGAAA | AACAACCACATCACTCTCAAC | (GGA)6 | 110 | 121132-121242 |
|  | FANhyb_64.1-1 | GAGGACATCATTGAATCAGAA | AAACTGTTCTCCAGACACTCA | (TG)7 | 114 | 121488-121602 |
|  |  |  |  |  |  |  |
| FANhyb_rscf00000682.1 | FANhyb_682.1-1 | AGTTTCCATGGGCTTGACCG | AGCACTATTCATTCAGTGCGT | (GAAA)3 | 440 | 22961-23400 |
|  | FANhyb_682.1-2 | ATTCTCCCAACTCTCACCTC | TACTCCACTGCTTTTGGATT | (CTG)4 | 104 | 27123-27227 |
|  | FANhyb_682.1-3 | CGGCTAAACACATTGAAACGGA | CAAACGAGGAATGCAGCTCG | (TAAA)3 | 264 | 28556-28822 |
|  |  |  |  |  |  |  |
| FANhyb_rscf00000045.1 | FANhyb_45.1-5 | AGGCCAAGACACACATATGGA | GCCCTTCTAATGAGGGATCCC | (AT)18 | 333 | 108080-108413 |
|  | FANhyb_45.1-4 | TTACAATCCTCCCTTTCTTTC | GATACATGCAGATGTGCTTTC | (AT)18 | 151 | 114479-114630 |
|  | FANhyb_45.1-2 | GACCTAGAACCCTTCTGTGTT | GGATAGACCAGAACTTCCTTC | (AG)11 | 141 | 115586-115727 |
|  | FANhyb_45.1-3 | CTTTGAATTCCTTCTTGGATT | ACTCACATAGGTCTGGAGTCA | (TCGA)3 | 150 | 115680-115830 |
|  | FANhyb_45.1-1 | GTTGCTGAAAGTCATTGAATC | AACCACAGCATCAAACTAAGA | (TC)10 | 155 | 131291-131446 |
