# Supplementary material for: Evidence of epistatic suppression of repeat fruiting in cultivated strawberry
Source: BMC Plant Biol. 2019 Sep 5;19:386. doi: 10.1186/s12870-019-1984-7 (PMC6729047; doi:10.1186/s12870-019-1984-7)
Supplement: Supplementary file 2 — Table S2. Amplification products from SSR markers in ‘Delmarvel’ × ‘Selva’ and ‘Tribute’ × ‘Honeoye’ strawberry (F. ×ananassa) F1 mapping populations (DOCX 24 kb) [file 12870_2019_1984_MOESM2_ESM.docx]

Table S2 Amplification products from SSR markers in ‘Delmarvel’ × ‘Selva’ and ‘Tribute’ × ‘Honeoye’ strawberry (*F*. ×*ananassa*) F_1_ mapping populations

|  | ‘Delmarvel' × ‘Selva’ | | | |  | 'Tribute’ × ‘Honeoye’ | | | |
| --- | --- | --- | --- | --- | --- | --- | --- | --- | --- |
| SSR | No. of products amplified | No. of products fit segregation | HG mapped | Product size linked to repeat-fruiting |  | No. of products amplified | No. of products fit segregation | HG mapped | Product size linked to repeat-fruiting |
| ANhyb19.1-1 | 4 | 2 | IV | 251-S^1^ |  | 4 | 2 | IV | 251-TH |
| FANhyb_19.1-2 | 3 | 3 | IV | None |  | 2 | None | - | - |
| FANhyb19.1-3 | 5 | 2 | IV | 442-S |  | 4 | None | - | - |
| FANhyb19.1-4 | 3 | 2 | IV | 189-S |  | 5 | 4 | IV | 189-T |
| FANhyb_19.1-5 | 4 | 2 | IV | None |  | NP^2^ | - | - | - |
| FANhyb-19.1-6 | 5 | 2 | IV | 213-S |  | 6 | 3 | IV | 213-T |
| FANhyb_19.1-7 | NA | - | - | - |  | NA | - | - | - |
| FANhyb_19.1-8 | NA | - | - | - |  | NA | - | - | - |
| FANhyb_19.1-9 | NA | - | - | - |  | NA | - | - | - |
| FANhyb_19.1-10 | NP | - | - | - |  | NP | - | - | - |
| FANhyb_19.1-11 | NP | - | - | - |  | NP | - | - | - |
| FANhyb_19.1-12 | NA | - | - | - |  | NA | - | - | - |
| FANhyb_19.1-13 | NP | - | - | - |  | 2 | 1 | IV | None |
| FANhyb_19.1-14 | NP | - | - | - |  | NP | - | - | - |
| FANhyb19.1-15 | 4 | 3 | IV | 121-S |  | 5 | 2 | IV | 121-T |
| FANhyb_19.1-16 | NP | - | - | - |  | NP | - | - | - |
| FANhyb_19.1-17 | 2 | 2 | IV | None |  | 4 | None | - | - |
| FANhyb_94.1-1 | NP | - | - | - |  | NP | - | - | - |
| FANhyb_611.1-1 | 2 | 1 | II | - |  | NP | - | - | - |
| FANhyb_611.1-2 | NP | - | - | - |  | NP | - | - | - |
| FANhyb_758.1-1 | 4 | 2 | VI | - |  | - | - | - | - |
| FANhyb_758.1-2 | NP | - | - | - |  | NP | - | - | - |
| FANhyb_758.1-3 | NA | - | - | - |  | NA | - | - | - |
| FANhyb_758.1-4 | 3 | 1 | VI | - |  | - | - | - | - |
| FANhyb_682.1-1 | NP | - | - | - |  | NP | - | - | - |
| FANhyb_682.1-2 | 1 | 1 | II | - |  | NP | - | - | - |
| FANhyb_682.1-3 | NP | - | - | - |  | NP | - | - | - |
| FANhyb_45.1-1 | 5 | 2 | I, II | - |  | - | - | - | - |
| FANhyb_45.1-2 | 7 | 3 | II | - |  | - | - | - | - |
| FANhyb_45.1-3 | NP | - | - | - |  | NP | - | - | - |
| FANhyb_45.1-4 | NP | - | - | - |  | NP | - | - | - |
| FANhyb_45.1-5 | NA | - | - | - |  | NA | - | - | - |
| FANhyb_64.1-1 | NP | - | - | - |  | - | - | - | - |
| FANhyb_64.1-2 | 5 | 3 | I | - |  | - | - | - | - |
| FANhyb_64.1-3 | NP | - | - | - |  | NP | - | - | - |
| FANhyb_64.1-4 | 4 | 2 | I | - |  | - | - | - | - |
| Bx052 | 7 | 1 | - |  |  | 6 | 4 | IV | None |
| Bx056 | 7 | 4 | IV | 227-S |  | 7 | 3 | IV | 227-T |
| Bx059 | 10 | 8 | IV | 250-S, 378-S |  | 10 | 5 | IV | 378-T |
| Bx064 | 10 | 8 | IV | 238-S, 240-S |  | 8 | 6 | IV | 238-T |
| Bx083 | 2 | 1 | IV | None |  | 3 | 3 | IV | None |
| Bx089 | 11 | 5 | IV | 236-S |  | 8 | 4 | IV | 236-T |
| Bx215 | 7 | 5 | IV | 148-S |  | 8 | 6 | IV | 148-T |
| Bx250 | 5 | 2 | IV | 242-S |  | 5 | None | - | - |

^1^D, S, T and H: product sizes (in base pair) amplified from ‘Delmarvel’, ‘Selva’, ‘Tribute’ and ‘Honeoye’, respectively

^2^NA: no amplification; NP: not polimorphic
